# Religion, faith, and spirituality influences on HIV prevention activities: A scoping review

**DOI:** 10.1371/journal.pone.0234720

**Published:** 2020-06-16

**Authors:** Vivian Vigliotti, Tamara Taggart, Mahaya Walker, Sasmita Kusmastuti, Yusuf Ransome

**Affiliations:** 1 Robbins Institute for Health Policy & Leadership, Baylor University, Waco, Texas, United States of America; 2 Department Social and Behavioral Sciences, Yale School of Public Health, New Haven, Connecticut, United States of America; 3 Prevention and Community Health, George Washington University School of Public Health, Washington, DC, United States of America; 4 Department of Public Health, University of Copenhagen, Copenhagen, Kobenhavns, Denmark; Ohio State University, UNITED STATES

## Abstract

**Introduction:**

Strategies to increase uptake of next-generation biomedical prevention technologies (e.g., long-acting injectable pre-exposure prophylaxis (PrEP)) can benefit from understanding associations between religion, faith, and spirituality (RFS) and current primary HIV prevention activities (e.g., condoms and oral PrEP) along with the mechanisms which underlie these associations.

**Methods:**

We searched PubMed, Embase, Academic Search Premier, Web of Science, and Sociological Abstracts for empirical articles that investigated and quantified relationships between RFS and primary HIV prevention activities outlined by the United States (U.S.) Department of Health and Human Services: condom use, HIV and STI testing, number of sexual partners, injection drug use treatment, medical male circumcision, and PrEP. We included articles in English language published between 2000 and 2020. We coded and analyzed studies based on a conceptual model. We then developed summary tables to describe the relation between RFS variables and the HIV prevention activities and any underlying mechanisms. We used CiteNetExplorer to analyze citation patterns.

**Results:**

We identified 2881 unique manuscripts and reviewed 29. The earliest eligible study was published in 2001, 41% were from Africa and 48% were from the U.S. RFS measures included attendance at religious services or interventions in religious settings; religious and/or spirituality scales, and measures that represent the influence of religion on behaviors. Twelve studies included multiple RFS measures. Twenty-one studies examined RFS in association with condom use, ten with HIV testing, nine with number of sexual partners, and one with PrEP. Fourteen (48%) documented a positive or protective association between all RFS factors examined and one or more HIV prevention activities. Among studies reporting a positive association, beliefs and values related to sexuality was the most frequently observed mechanism. Among studies reporting negative associations, behavioral norms, social influence, and beliefs and values related to sexuality were observed equally. Studies infrequently cited each other.

**Conclusion:**

More than half of the studies in this review reported a positive/protective association between RFS and HIV prevention activities, with condom use being the most frequently studied, and all having some protective association with HIV testing behaviors. Beliefs and values related to sexuality are possible mechanisms that could underpin RFS-related HIV prevention interventions. More studies are needed on PrEP and spirituality/subjective religiosity.

## Introduction

An estimated 40 million people are living with HIV (PLWH) and the number of people newly HIV infected declined by a modest 10% between 2013 and 2017 [1]. Approximately 40% of PLWH are not accessing antiretroviral therapy (ART); therefore, they miss opportunities to improve their quality of life and lower transmission risk in the population [2, 3]. To halt transmission and reduce HIV incidence, combination approaches—those that integrate biomedical, behavioral, and structural factors, are necessary [4]. Currently, biomedical technologies in the form of antiretroviral drugs have been dominating the discourse both for secondary treatment as prevention (TasP) promoted through campaigns such as “Undetectable = Untransmittable” (U = U) [5] and primary prevention activities such as Pre-Exposure Prophylaxis (PrEP) [6, 7]. However, for those biomedical modalities to be effective and achieve maximum population impact; individuals need to maintain high levels of adherence [8], which requires attention to cultural context [9] and who is being targeted [10] because ART can be used for either prevention or treatment [11].

Beyond cost and other structural barriers such as availability, one’s ability to achieve high levels of adherence are largely affected by one’s social circumstances [12]. We know that norms within cultural contexts either constrain or empower one’s agency to engage in HIV prevention behaviors [13–15]. Therefore, to reduce HIV incidence through combination prevention approaches, we need to understand the influence of upstream social and cultural factors including norms, values, networks, structures, and institutions [16, 17]. However, to date, we know little about effectiveness and impact of social and cultural interventions on reducing HIV burden and forward transmission [18].

We conducted this study, therefore, to investigate the role of religion, faith, and spirituality (RFS) on primary HIV prevention. Religion is one key social and cultural factor [19–21] with pervasive influence over the norms, values, structures, and institutions, which profoundly impact individuals’ behaviors and decisions [22, 23]. Religion can be defined broadly as a system of symbols, rites, experiences, and rituals that have powerful, pervasive, and long-lasting actions and motivations that are generated through concepts of existence, which captures awe and dependence of a power greater than one’s self [24, 25]. Religion is timely to study and imperative to include in any combination of HIV prevention activity [26]. Approximately 84% of the world’s population self-report being religiously affiliated [27]. Moreover, the countries with the fastest growing population, including those with highest HIV burden, report high religious involvement [28]. For instance, in South Africa, the region with highest HIV prevalence in the world; 56% of the population report being active members of church or religious organizations [29]. Faith is another construct, that when applied to religion describes a psychological cognitive process of developing a system of knowing, giving coherence to life, and valuing [30]. Spirituality is another construct that overlaps but is distinct from religiosity. It has been defined as one’s personal experience, belief or relationship with a divine/higher power or search for the sacred, where the sacred may or may not be connected to religion [31–33].

We study primary HIV prevention because of the increasing trend towards and rapid availability of biomedical prevention options such as long-acting injectable PrEP, MK-8591-eluting implant, and dolutegravir (DTG)-based HIV treatment during pregnancy. Therefore, we need to better understand and activate social and cultural factors that could expedite uptake and adherence of these technologies. We also focus on primary prevention because prior review studies of religion and HIV already covered health and HIV-clinical outcomes *among* PLWH such as viral suppression, CD4+ count, and ART adherence [34, 35]. Next, there is only one published systematic review on religion and primary HIV prevention, however that study narrowed in on sexual HIV risk behaviors such as sexual initiation and sexual experience [36]. The consensus from those studies was that religious factors are mostly protective. However, several gaps in knowledge remain, which our study aims to fill.

First, we provide evidence of the association between RFS and other key primary HIV prevention activities outlined by the United States Department of Health and Human Services (U.S. DHHS): condom use, HIV and sexually transmitted infection (STI) testing, reducing number of sexual partners, injection drug use treatment, medical male circumcision, and PrEP [37]. Second, we identify potential mechanisms which underlie both positive and negative associations. Clarifying these mechanisms may inform HIV prevention interventions and implementation science activities worldwide. Third, we provide a fuller description of “religion’s” impact on HIV by expanding or refining operational definitions to include constructs such as faith and spirituality, given these are sometimes conflated yet may have different implications for interventions.

## Methods

### Search strategy

We searched five databases—PubMed, Embase, Academic Search Premier, Web of Science, Sociological Abstracts—using predefined keywords (S1 Table) developed in consultation with our university librarian. We conducted the search in accordance to the PRISMA Statement, for articles published between January 1, 2000 and February 20, 2020. We submitted our protocol to PROSPERO (S1 Document) at the beginning of our study, however, it was not registered since scoping reviews are not considered.

### Study selection

Our inclusion criteria were articles: (1) written in English; (2) published between January 1, 2000 and February 20, 2020; (3) peer-reviewed; (4) considered an HIV prevention activity and RFS; and (5) quantified the association between RFS and the HIV prevention activities (S2 Table). First, two authors (VV, MW) independently reviewed the title and abstract and then the full-text of each article, with a third author (YR) involved to resolve conflicts. Next, two authors (VV, MW) extracted data from the included articles. We (VV, YR, TT) further excluded articles during the extraction phase, after closer scrutiny against the inclusion criteria. Senior author (YR) conducted a final round of exclusions during the analysis phase. The selection process resulted in 29 final articles for inclusion.

### Quality assessment

One reviewer (TT) conducted a quality assessment of the 29 included studies using a checklist for assessing quality in observational studies [38]. Six domains were used to assess risk of bias: 1) methods for selecting study participants, 2) methods for measuring independent and dependent variables, 3) design-specific source bias, 4) method for controlling confounding, 5) statistical methods, and 6) other biases including conflict of interest and disclosure of funding sources. We scored each study as low, high, or unclear for risk of bias for each domain [39].

### Data extraction

#### Religion, faith, spirituality

We identified pre-specified categories for the RFS measures used in Shaw and El-Bassel [36] and expanded the operational definitions of those categories based on theoretical frameworks of religion, spirituality and health [33, 40]. This process resulted in four RFS categories. The first category is service attendance, which we operationalized as studies that measured individuals’ frequency of attending religious or spiritual services or studies where there was an intervention that involved attending sessions within an RFS setting. The intervention could be RFS-based or secular. However, for measures that included attendance as part of a longer composite index, we did not include it in the attendance category because we could not isolate its unique impact. The second category is religious scales, which includes studies that used validated scales from prior literature (e.g., The Duke University Religion Index (DUREL) [41] or a new scale the authors created using psychometric methodologies (e.g., prevalence of religious beliefs discouraging homosexuality in society) [42].

The third category is a combination of spirituality and subjective religiosity measures. Spirituality characterizes one’s personal experience or relationship with God or a higher power [43]. Spirituality measures may also include indicators of subjective religiosity, which defines experiences unique to the individual that are not directly observable (e.g., self-rated importance of God in one’s daily life) [44, 45]. The fourth category is the influence of religion on behaviors. This category was developed to account for studies that do not include service attendance or spirituality measures, but rather include a global belief system perspective.

#### Religious denomination

In addition to those four categories, we organized data according to the broad typologies of religious traditions/denominations used in Shaw and El-Bassel (2014) and others identified through the abstract review stage. The categories included: Catholic; Muslim; Protestant (because not all studies permitted distinguishing the three major branches); Christian (for those that did not specify tradition but examined Judeo-Christian aspects); and other, which included studies that did not necessarily mention denomination or others that fell outside of the prior categories (e.g., Buddhism). We extracted and coded denomination *only* from quantitative studies where we could compare the association with an HIV prevention activity across one or more denomination. In qualitative studies, we coded denomination when we could examine differences in text according to the denomination. Studies were not included if they were conducted among one or more of these groups but did not provide information that facilitated comparisons.

#### Mechanisms

We classified studies by the mechanisms that potentially connect the associations between RFS measures and the HIV prevention activities. We began with categories used in Shaw and El-Bassel (2014) then added other established behavioral and psychosocial pathways [40, 46] or new pathways identified during review (e.g., beliefs and values). This process resulted in seven mechanisms. We identified mechanisms in quantitative studies through methods such as mediation analysis, multiple adjustment or partial correlations, or from the text if the authors described factors not presented in tables. In qualitative or mixed-methods studies, we identified mechanisms through blocks of text if they discussed examples that explained their findings.

The first mechanism is behavioral norms, which describes the use of religious doctrine to regulate behaviors (e.g., Old Testament laws that prohibit pre-marital sex). The second mechanism is social organization and social support, which describes the features of religious institutions (e.g., having a health ministry) and other types of non-tangible support that influences behavior. The third mechanism is social influence, which describes the degree of regulating one’s behavior by virtue of belonging to a religious congregation or faith tradition, but not necessarily tied to a religious doctrine (e.g., self-regulation from identifying as Christian). Fourth is education, which includes information delivered directly through faith institutions and ministries, directly from faith leaders, clergy, or knowledge shared among parishioners. Fifth is beliefs and values related to sex and sexuality, which deals specifically with issues of sex, love, and marriage between people of the same gender. The sixth mechanism is circumcision. The seventh mechanism is alcohol use, which we identified as a unique category given its relationship to both religion and HIV [47, 48].

#### Direction of association

We coded direction of association in quantitative studies based on coefficients and in qualitative studies based on interpreting passages of texts. We identified four categories: (1) positive/protective, (2) negative, (3) mixed findings (i.e., either positive and negative associations) in the same study, and (4) neutral, not significant or insufficient information to qualitatively determine a direction. Mixed findings in the same study could indicate either one RFS measure was significantly associated with one HIV prevention activity but not another or that some RFS measure have significant or different directions of association with the same HIV prevention activity. In qualitative studies, this indicated that some factors were viewed positively for one HIV prevention activity but negatively for others.

### Data synthesis and analysis

Reviewers (YR and TT) reviewed for consensus, the categories of the RFS categories and the mechanisms then compiled the studies into Stata 15.0 software for data management and descriptive analysis. Reviewer (YR) conducted descriptive analysis based on the study’s conceptual model, and (YR and TT) developed the tables.

### Secondary exploratory analyses

One of the secondary aims of this review was to characterize how studies of RFS and HIV prevention are informed by prior studies. There is significant variation in study designs and measurement of RFS constructs which may inhibit the potential to obtain consensus about the impact of religion in HIV prevention. We downloaded the full record contents of the 29 included articles from our primary search on Web of Science Core Collection database, as well as all secondary articles that cited or were cited by the primary articles. We then performed analyses and visualizations on citation patterns in the scientific literature using the CiteNetExplorer software.

### Ethical consideration

The study did not require IRB review since it does not involve human subjects.

## Results

### Descriptive study characteristics

**Fig 1:**
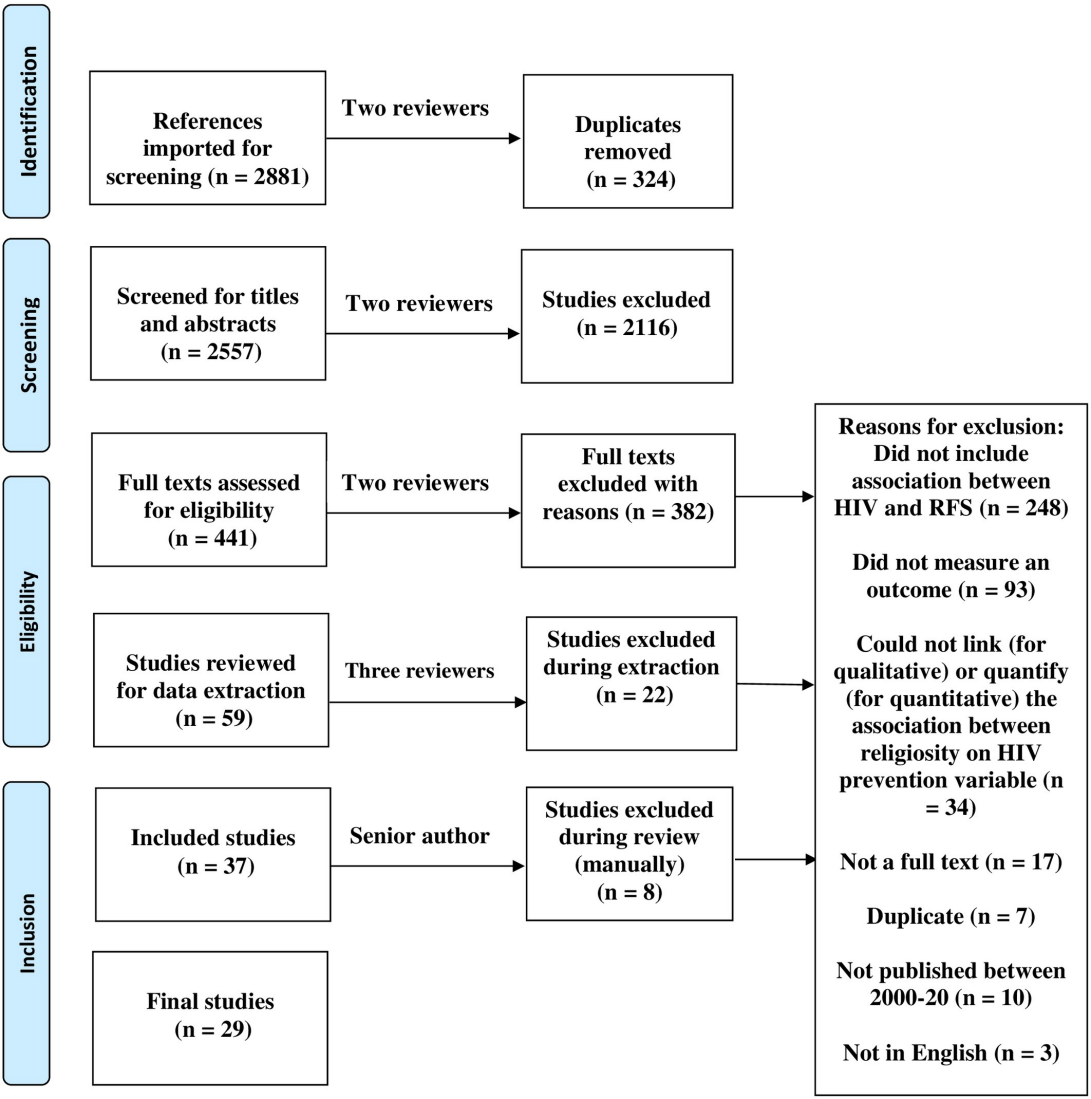
PRISMA flow-chart of included articles (n = 29). Among 2881 articles, approximately one percent (n = 29) were met criteria and included for this review. Among the full-texts that were assessed for eligibility (n = 441), the majority (56%, n = 248) were excluded because they did not allow us to assess the association between religion, faith, and spirituality with an HIV prevention variable.

We screened 2557 titles and abstracts after duplicates were removed and reviewed 441 for full text, then included 29 studies for data extraction. We excluded studies when they did not assess RFS or did not include one of the HIV prevention activities (Fig 1).

### Quality assessment

Among the 29 selected studies, 14 were at low risk for all 6 methodological quality items. The remaining studies (n = 15) had at least one of the bias items and none of the selected studies were at high risk for all the bias items (S3 Table).

Descriptive information for the 29 studies is in Table 1 and (S4 Table) for more details. While not all studies reported relevant information such as age, gender, etc., percentages are based on all the studies. The first study was published in year 2001 and four (14%) were published in year 2013. Twelve studies (41%) were based on samples from Africa and fourteen (48%) on samples from the U.S. Seventeen studies (59%) were quantitative, one qualitative, and eleven (37%) were mixed methods. The sample size of individuals studied ranged from 43 to 5534 with a mean of 1136 (SD = 1187). The ages of the individuals across the studies ranged between 12 and 80 years of which six (21%) were among individuals 12–24 years. Twenty-one studies (72%) included both men and women. Twelve studies (41%) included only one RFS measure and 12 that use multiple RFS measures. For the HIV prevention activities, the studies are not mutually exclusive, and percentages do not always sum to 100. Twenty-one studies examined condom use, ten studies examined HIV testing, and nine with number of sexual partners. Only one study examined the association with PrEP during our review period.

**Table 1:** Selected characteristics of the studies included in the review (n = 29), sorted by ascending publication date.

Reference number	Publication date	Location (country)	Sample (gender, age, race, marital status)	Sample size	Design (quantitative = 1; qualitative = 2; mixed = 3)	Condom use	HIV testing	STI testing	Number of sexual partners	Injection drug use	Pre-exposure prophylaxis (PrEP)	Male circumcision
Avants, et al.	2001	US	M/F	43	3					X		
McCree, et al.	2003	US	F, 14–19, B, NM	522	1	X						
Agadjanian	2005	Africa	M/F, B	731	3	X						
Margolin, et al.	2006	US	M/F, 21–56, W/B/H	72	1	X				X		
Agha, et al.	2006	Africa	M/F, 13–20	5534	1	X						
Cerqueira-Santos, et al.	2008	Brazil	M/F, 12–24	1013	1	X						
Perez-Jimenez, et al	2009	Puerto Rico, Dominican Republic, Mexico	M/F, 18–62, B/H, M/NM	94	3	X			X			
Coleman, et al.	2009	US	M, 40–68, B	130	1	X						
Trinitapoli, et al.	2009	Africa	M, 15–80	1500	3	X						
Wu, et al.	2010	China	M/F, 15–60, A	2624	1	X						
Agardh, et al	2010	Africa	M/F	980	1	X			X			
Berkeley-Patton, et al	2010	US	M, 35–44, B/W	3200	3		X					
Agardh, et al	2011	Africa	M/F, B	1220	1	X			X			
Muula, et al.	2011	Africa	F, 25–43, B, M	1664	3	X	X					
Trinitapoli, et al	2011	Africa	F, B	187*	3	X	X					
Mash, et al.	2012	Africa	M/F, 12–20, W/B/H	1600	3	X			X			
Wingood, et al.	2013	US	F, 18–34, B, NM	134	1	X						
Szaflarski, et al	2013	US	M/F, 18+, W/B/H	447	1		X					
Kagimu, et al	2013	Africa	M/F, 15–24	1224	1	X			X			
Downs, et al.	2013	Africa	M/F	67	2	X						X
Eriksson, et al.	2014	Africa	M/F, 15–24	1102	3		X		X			
Ezeanolue, et al.	2015	Africa	F, 16+, M/NM	2700	1		X					
Stewart, et al.	2016	US	M/F, B, 18–57	71	3	X						
Derose, et al.	2016	US	F, 18+, B/H	1235	3		X					
Nelson, et al.	2017	US	M, 18+, B/H	1553	1	X			X			
Williams, et al	2018	US	M/F	1306	1		X					
Ransome, et al	2018	US	M/F, B	868	1		X				X	
Berkley-Patton, et al	2019	US	M/F, B, 18–64	543	1		X		X			
Jemmott, et al	2020	US	M/F	613	1	X			X			

* indicates that the sample was congregations whereas other sample sizes refer to individuals.

Twelve studies included measures of religious service attendance. Five included spirituality/subjective religiosity scales in whole or modified, and thirteen included the influence of religion on behavior. Among the studies where we could compare the association across denominations/religious traditions, Catholic (n = 8) and Christian denominations (n = 7) were the most frequently included. Fourteen studies (48%) reported a positive/protective direction of association between the RFS variables they examined and an HIV prevention activity; three (12%) reported negative associations, and eight (27%) mixed (both positive & negative associations in the same sample). Four studies (14%) contained findings that were neutral, not significant or insufficient information to qualitatively determine a direction of association.

### Summary from statistical analysis

#### Direction of associations

The following results are from statistical analysis guided by our conceptual model (S1 Fig). We observed a broad trend for HIV testing where 100% of the studies that examined religious service attendance and influence of religion on behavior were positive/protective. Next, five of eight (62%) of the studies that examined service attendance and condom use were positive/protective. The one study that examined PrEP also found a significant positive association (Table 2).

**Table 2:** Summary of the direction of associations for the religion, faith, and spirituality in association with HIV prevention activities.

	Condom use		HIV/STI testing		Number of sexual partners		Injection drug use		Pre-exposure prophylaxis (PrEP)	
Direction of association (% of studies within each HIV prevention variable)										
	Positive	Negative	Positive	Negative	Positive	Negative	Positive	Negative	Positive	Negative
**Religious service attendance**	5/8	1/8	5/5	0/5	2/4	0/4	1/1	0/1	1/1	0/1
**Religion and/or spirituality scale**	2/4	0/4	0/1	0/1	1/2	0/2	0	0	0	0
**Spirituality/ Subjective religiosity**	0	0	0	0	0	0	1/1	0/1	0	0
**Influence of religion on behavior**	4/11	1/11	2/3	0/3	1/6	0/6	0	0	1/1	0/1

n = number of studies for that outcome and the specific religion item assessed. The studies are not mutually exclusive so one study could have assessed religious service attendance and spirituality with condom use. Therefore, the denominator of studies from which each percentage is derived may vary. For example, religious service attendance and condom use (n = 8) whereas influence of religion on behavior and condom use (n = 11). The directions of associations, however, are exclusive where (1 = positive only, 2 = negative only, 3 = mixed (both positive and negative in the same sample), and 4 = neutral, not significant or insufficient information to qualitatively determine a direction). The latter two categories are excluded from this table. Positive association describes a protective relationship.

#### Mechanisms

This section describes patterns of common mechanisms stratified by studies reporting positive/protective and then negative associations between RFS and HIV prevention activities (Table 3). First, among the studies that documented a positive association; social influence and beliefs and values related to sexuality were the primary mechanisms identified, and these were most represented for condom use and number of sexual partners. For HIV testing, social influence and education were the most often identified mechanisms. Second, among these studies, mechanisms were mostly identified when service attendance or influence on behavior was the focal RFS measure. One that examined condom use documented a negative association with RFS. Mechanisms identified from that one study included behavioral norms, beliefs and values related to sex and sexuality, and social influence. There were no studies among the other HIV prevention activities that documented a significant negative association. Lastly, we found no evidence of alcohol use as a mechanism linking religion and HIV prevention activities.

**Table 3:** Top two mechanisms among studies that document a positive/protective or negative association between religious and spiritual measures and HIV prevention variables.

	Condom use	HIV/STI testing	Number of sexual partners	Injection drug use	Pre-exposure prophylaxis (PrEP)
	**Mechanism, among studies finding a positive/protective association**				
Religious service attendance	Beliefs and values related to sex and sexuality tied with Social influence.	Education tied with Social influence, and Beliefs and values related to sex and sexuality.	Beliefs and values related to sex and sexuality, and social influence.	N/A	Social influence, and Beliefs and values related to sex and sexuality.
Religion and/or spirituality scale	Social influence and Beliefs and values related to sex and sexuality.	Behavioral norms, Social influence, Education, and Beliefs and values related to sex and sexuality.	Behavioral norms, Social influence, Education, and Beliefs and values related to sex and sexuality.	No studies	No studies
Spirituality/ subjective religiosity	No studies	No studies	No studies	N/A	No studies
Influence of religion on behavior	Social influence, followed by a tie between Social organization and support and, Beliefs and values related to sex and sexuality.	Social influence, Education, and Social organization & support.	Beliefs and values related to sex and sexuality, and Social influence.	No studies	Social influence, and Beliefs and values related to sex and sexuality.
**Mechanism, among studies finding a negative association**					
Religious service attendance	Behavioral norms and, Beliefs and values related to sex and sexuality.	No studies	No studies	No studies	No studies
Religion and/or spirituality scale	No studies	No studies	No studies	No studies	No studies
Spirituality/ subjective religiosity	No studies	No studies	No studies	No studies	No studies
Influence of religion on behavior	Social influence	No studies	No studies	No studies	No studies

N/A means that it was not possible to identify mechanisms in the studies either because no mechanisms were reported, or the research methods did not allow us to disentangle the mechanisms from the primary exposure in the specific category. No studies means there was none in that category to evaluate, and the number of studies can be seen in Table 2. More than one mechanism may be present in the same study and so the mechanism may not be present across all studies in that category.

**Behavioral norms**: use of religious doctrine to regulate behaviors (e.g., Old Testament laws that prohibit pre-marital sex).

**Social organization and support**: the features of religious institutions (e.g., having a health ministry) and other types of non-tangible support that influences behavior.

**Social influence**: the degree of regulating one’s behavior by virtue of belonging to a religious congregation or faith tradition, but not necessarily tied to a religious doctrine (e.g., self-regulation from identifying as Christian).

**Education**: information delivered directly through faith institutions and ministries, directly from faith leaders, clergy, or knowledge shared among parishioners.

**Beliefs and values related to sex and sexuality**: studies dealing directly with issues of sex, love, and marriage between people of the same gender.

### Expanded details from statistical analysis

The following results section provides greater detail on the RFS measures and the HIV prevention activities and mechanisms involved according to our conceptual model.

#### Condom use

*Attendance*. Eight studies examined condom use in association with service attendance [49–56]. Five of those eight studies (62%) documented a positive/protective association and were statistically significant [49, 51, 52, 54, 56]. The most frequent mechanism reported among studies that documented a protective association was beliefs and values related to sex or sexuality and social influence. One study [53] reported a negative association with condom use. They found that, among a sample of persons attending Mainline Protestant Churches in Maputo and Chibuto, Mozambique, those who attended church three times or more per week had 47% lower odds of using condoms with a partner compared to other prevention measures. Behavioral norms, and beliefs around sex and sexuality were the primary mechanisms identified.

*Religion and/or spirituality scale*. Four quantitative studies examined condom use in association with religious or spiritual scales [42, 57–59]. Most of these studies reported some psychometric properties such as Cronbach’s alpha. In other studies, the authors created new scales using scale creation methods such as factor analysis. The authors gave the scales their own names such as “religiosity,” “religiosity/spirituality,” and “prevalence of discouraging homosexuality beliefs” [42, 57, 59].

Two studies documented a positive/protective statistically significant association [58, 59]. One example is Coleman and Ball (2009), who analyzed the Spiritual Well-Being Scale among a sample of middle aged African American HIV-infected men. They found that higher religious well-being was associated with higher self-efficacy to use condoms. Social influence and beliefs related to sex and sexuality were identified as the primary mechanisms in that study. No studies documented significant negative associations and some studies were null or could not be determined.

*Spirituality/subjective religiosity*. There were no studies in this category.

*Influence of religion on behavior*. Eleven studies examined condom use in association with the influence of religion on behavior [42, 49, 50, 54–56, 60–64]. These studies were mostly mixed methods (n = 7). One example was Berkeley-Patton, Bowe-Thompson et al. 2010, a mixed-methods study where approximately 50% of participants reported incorrect responses about the proper way to use condoms. After asking participants how they could explain the low proportion of correct responses, they said that several pastors believed that the church was not the most appropriate setting to discuss condoms. Four of the eleven studies reported a positive/protective statistically significant association, or for the qualitative studies, mentioned religion directly as a factor [49, 54, 56, 64]. For example, in Trinitapoli 2009, pastors privately advising condom use was significantly associated with higher condom use. Social influence, social support and organization, education, and beliefs and values related to sex and sexuality were identified with similar frequency within the studies. One study found a negative association, and social influence was the primary mechanism. One other study had mixed directions where influence on behavior was not associated with condom use but was significantly associated with another HIV prevention activity.

#### HIV/STI testing

*Attendance*. Five studies examined HIV/STI in association with service attendance [49, 65–68]. Two studies were mixed methods and three were quantitative. Positive/protective associations were observed for all studies. For instance, in Ezeanolue, Obiefune et al. 2015, participants in an intervention group who received health education at baby showers held in churches (compared to control group churches) had higher adjusted odds of HIV testing post-intervention. Berkley-Patton, Thompson, and Moore et al. 2019 found significant increases in HIV testing at 12 months among participants who attended (59% vs 42%, p = 0.008) their Taking it to the Pews (TIPS) vs standard-information intervention. The most frequent mechanisms identified among studies reporting a positive/protective association were social organization/support, beliefs and values related to sexuality, and education.

*Religion and/or spiritual scale*. There was only one study in this category [67]. The authors assessed religiosity using a seven-item version of the Religious Background and Behavior Survey on participant’s engagement in church activities and one item on a description of their degree of religiosity. Religiosity was statistically unrelated to HIV testing in the multivariable model.

*Spirituality/ subjective religiosity*. There were no studies in this category.

*Influence of religion on behavior and HIV/STI testing*. Three studies met the criteria [49, 68, 69]. One mixed methods study examined HIV/STI testing in association with the influence of religion on behavior [49]. That study found a positive/protective association where encouragement from church members to get tested for HIV was significantly greater than encouragement from family and friends. Also, people exposed to religious teachings on HIV and stigma were more likely to get tested for HIV. Several mechanisms were present in those studies including behavioral norms, social support and organization, social influence, and education. One example of social support and organization and social influence was that 87% of participants in that study [49] believed it was important for their church to talk about testing for HIV and 77% reported that the church should offer HIV testing.

#### Number of sexual partners

*Attendance*. Four studies examined the number of sexual partners in association with our definition of religious service attendance [50, 54, 55, 67]. No mechanisms were identified. Those studies did not find a statistically significant relationship between the variables.

*Religion and/or spiritual scale*. Two studies examined the number of sexual partners in association with religion and/or spiritual scale [42, 67] but the associations were not significant in those studies.

*Spirituality/ subjective religiosity*. There were no studies in this category.

*Influence of religion on behavior*. Six studies examined the number of sexual partners in association with the influence of religion on behavior [42, 50, 54, 55, 60, 61]. Five were quantitative and one was mixed methods. Only one found a significant positive relationship with this outcome. In the study that found the positive relationship, one measure of influence on behavior was “trying hard to implement religious teachings.” Those with high influence (i.e., participation in religious activities multiple times a day) compared to lower influence had higher odds of having one sexual partner [54].

#### Injection drug use

*Attendance*. Only one study examined injection drug use in association with religious service attendance [51]. This quantitative study, conducted among a sample of methadone clients, reported a positive association.

*Religion and/or spiritual scale*. There were no studies in this category.

*Spirituality/ subjective religiosity*. One study examined injection drug use in association with spirituality/subjective religiosity [70] and found that higher mean spiritual support ratings was associated with a higher number of weeks not using illicit drugs. No mechanisms were identified.

*Influence of religion on behavior*. There were no studies in this category.

#### Pre-exposure prophylaxis (PrEP)

There was only one published study in this category [68]. The study found that persons who attended religious services a few times a year (compared to those who never attended) had 50% higher odds of being willing to use PrEP. They also found that participants who reported hearing faith leader’s messages about same-sex relationships were also willing to use PrEP.

#### Medical male circumcision

One qualitative study examined male circumcision in association with influence of religion on behavior [62]. They found a negative association, where 60% of participants worried that promoting circumcision in church would increase promiscuity. We identified social influence and behavioral norms as mechanisms.

#### Comparisons across religious denominations

Eight studies included denomination, but only six studies were structured to facilitate direct comparisons across religious denominations, so two were excluded [71, 72]. There was significant variation in the categorization of denominations, the number included, and the HIV prevention variable that was compared although most examined condom use [53, 56, 73–75] and one also examined HIV testing [76]. Significant differences were observed in all but one study. For example, in Agadjanian 2005, the impact of attending services three or more times a week on condom use was protective (OR = 0.47) for participants in mainline churches compared to those in “healing churches,” (OR = 0.97) (e.g., Apostolic & Pentecostals). In another study, healing churches tended to have lower mean messaging around condom use and HIV prevention compared to other denominations [73].

#### Citation analysis findings

**Fig 2:**
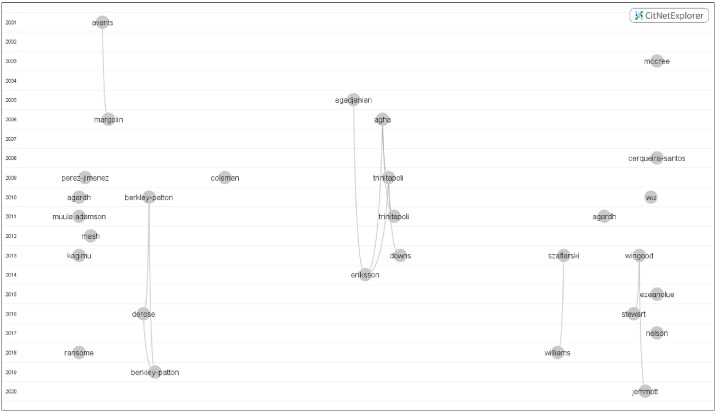
Citation analysis patterns among 29 included studies. Visualization of citation links among the 29 included studies, displayed according to timeline of publication year with more recent studies being located below older studies. Each circle represents a study labeled by the last name of the first author and curved lines represent direct citation links. Out of the 29 studies, ten studies directly cited one or more studies in the group and this could likely be a result of similar topics or concepts being examined.

Fig 2 shows a visualization of citation links among the 29 included studies, displayed according to a timeline of publication year, with more recent studies being located below older studies. Each circle represents a study labeled by the last name of the first author and curved lines represent direct citation links. If studies cited each other and published in the same year, then the citing study is always located somewhere below the corresponding cited study. The position of the study on the horizontal axis is determined by the closeness of publications in the citation networks; the closer the circles are to each other, the closer the studies are related to each other. Clear clusters were observed. Jemmott et al. 2020, the latest study, cited studies that were published from 2013 to present. Among the 29 included studies, five were in the top 100 most cited (S2 Fig). Berkley-Patton, Bowe-Thompson et al. 2010 was the highest cited among the 29 studies, followed by Agadjanian 2005, Trinitapoli 2009, McCree, Wingood et al. 2003, and Agha, Hutchinson et al. 2006. Only five of the included 29 studies also directly cited one or more authors, and this could be likely a result of similar topics or concepts being examined (S2 Fig).

## Discussion

This review provides a comprehensive assessment of the associations and mechanisms connecting religion, faith, and spirituality (RFS) constructs to primary HIV prevention activities (i.e., condom use, HIV and STI testing, PrEP) and discusses current research gaps and implications for future research and practice.

The first primary finding was that condom use was the most studied strategy in association with RFS and frequency of religious service attendance was the most assessed RFS factor. Our study confirmed what is already documented that RFS is often positively associated with several of the prevention outcomes and that degree of protective association varies. For instance, higher frequency of service attendance was positively associated with condom use in 62% of the studies in this review and positively associated with HIV testing in 100% of the studies included. Second, despite a few recent studies showing an association between RFS and willingness to use- and acceptability of- PrEP [77] and RFS, PrEP remains understudied [68]. Third, only one study examined the associations between spirituality and subjective religiosity and HIV prevention. This is a notable gap that should be addressed in future work on this topic in-light of evidence from the U.S. [78] that more people are identifying as spiritual but not religious [79]. One plausible explanation for this finding is that challenges related to conceptualizing and measuring these factors include tautological errors (e.g., correlating spirituality as positive psychological state and using it as a predictor of other psychological states). Another challenge is little consideration for other cultural and psychosocial factors, and subjectivity [80, 81].

The fourth major finding is related to mechanisms likely to link RFS and HIV prevention. Social influence was the most commonly identified mechanism among studies reporting a positive/protective association and was often present in studies that examined service attendance or used a religious and spiritual scale. The behavioral norms category was the most commonly reported pathway for studies reporting a negative association. These findings indicate that RFS-based HIV prevention interventions may benefit from including strategies that draw upon social norms, expectations, and networks. Other recent work suggests that non-fundamentalist theology (i.e., those not based on strict interpretations of the Bible and religious text) may be important for congregations to be engaged in HIV prevention activities [82]. The fifth finding is that, among the few studies that facilitated comparisons across religious denominations/traditions, there are significant differences in the size of associations. This finding, along with projected shifts in religious denominations in countries with a high HIV incidence [83], suggests that denomination should be assessed when determining the scalability and generalizability of results from RFS-based interventions. Sixth, we found that a limited number of studies were highly cited by other studies not included in this review. Further, among the 29 studies identified, even fewer authors cited each other. This limitation could be explained by differences in disciplines of research (e.g., sociology or public health) or cultural context. However, the lack of cross-citation of prior work potentially weakens the evidence base for interventions and diminishes consensus on common measures or operationalizations for RFS categories.

Our main study strengths, that builds upon a recent prior study [36], are that we identify the associations between RFS and a broader set of HIV prevention activities, and we assess mechanisms among studies that find both positive and negative associations. We also conducted citation analyses which, to date, has not been previously reported in the literature on RFS and HIV prevention. Our findings indicate that future studies may wish to draw from studies in different contexts and further in time. Some limitations are that we did not include an exhaustive set of prevention activities (e.g., Post-exposure Prophylaxis (PEP) or vaccines). Nevertheless, we captured the most prevalent HIV prevention activities [9]. Next, our RFS categories could potentially miss other religious traditions and practices, such as traditional healing or Hinduism. However, we used and tested a comprehensive search strategy and the RFS categories we used reflect the distribution of religious groups globally [83]. Moreover, the included studies are from regions (e.g., Asia and Africa) where these other traditions may be used. We did not consider grey-literature sources such as reports, since the quality and validity of these studies can be questionable, and methods might not contain sufficient details to be reproducible. Last, the mechanisms are not exhaustive, but we aimed to build upon prior work and identify others as they arose from our search.

## Conclusion

We found protective associations between RFS and some primary HIV prevention activities such as condom use and HIV testing. We recommend that future work should include other measures of HIV prevention strategies that align with Ending the HIV Epidemic goals, such as PrEP use and adherence. Studies should also include reliable and multidimensional measures of spirituality and subjective religiosity and assess denomination. More studies should include randomized controlled and quasi-experimental designs that better facilitate causal inference, mediation analyses that can statistically quantify potential mechanisms, and ethnographic methods to observe how RFS works in practice. Next, quantitative studies that investigate HIV stigma as a mechanism (e.g., mediator between RFS and some HIV prevention outcome) are needed since there is already several interventions tackling stigma [65, 84–86]. One recent study that evaluated an intervention “Love with no Exceptions” among clergy and church members in Alabama found very little change in HIV stigma attitudes before and after the intervention [87]. For current practice, interventionists can utilize RFS [88, 89] to address other known social and cultural factors such as stigma and discrimination [90], which are barriers to PrEP uptake [91, 92] and likely to also influence uptake of next generation biomedical technologies.

## Supporting information

S1 Table (DOCX)

S2 Table (DOCX)

S3 Table (DOCX)

S4 Table (DOCX)

S1 Fig (TIF)

S2 Fig (TIF)

S1 Document (PDF)

## Abbreviations

HIV: human immunodeficiency virus
IDU: injection drug use
PrEP: pre-exposure prophylaxis
RFS: Religion, faith, and spirituality
STD: sexually transmitted disease
STI: sexually transmitted infection
U.S.: United States
