# Correction: Religion, faith, and spirituality influences on HIV prevention activities: A scoping review

**DOI:** 10.1371/journal.pone.0241737

**Published:** 2020-10-28

**Authors:** Vivian Vigliotti, Tamara Taggart, Mahaya Walker, Sasmita Kusumastuti, Yusuf Ransome

The fourth author’s name is spelled incorrectly. The correct name is: Sasmita Kusumastuti. The correct citation is: Vigliotti V, Taggart T, Walker M, Kusumastuti S, Ransome Y (2020) Religion, faith, and spirituality influences on HIV prevention activities: A scoping review. PLoS ONE 15(6): e0234720. https://doi.org/10.1371/journal.pone.0234720.

## References

[1] Vigliotti V, Taggart T, Walker M, Kusmastuti S, Ransome Y (2020) Religion, faith, and spirituality influences on HIV prevention activities: A scoping review. PLoS ONE 15(6): e0234720 10.1371/journal.pone.0234720 32544212 PMC7297313

