# Supplementary material for: Religion, faith, and spirituality influences on HIV prevention activities: A scoping review
Source: PLoS One. 2020 Jun 16;15(6):e0234720. doi: 10.1371/journal.pone.0234720 (PMC7297313; doi:10.1371/journal.pone.0234720)
Supplement: S1 Table — (DOCX) [file pone.0234720.s001.docx]

| **Question** | **Yes** | **No** |
| --- | --- | --- |
| Is the article in English? |  |  |
| Is the article published between January 1, 2000 and February 20, 2020? |  |  |
| Is the article peer-reviewed? |  |  |
| Does the article consider HIV prevention and religion/spirituality/faith? |  |  |
| Is the effect of religion, faith organizations, faith, or spirituality on HIV prevention methods quantified? |  |  |

**Appendix Table 1. Decision Criteria**
