# Supplementary material for: Religion, faith, and spirituality influences on HIV prevention activities: A scoping review
Source: PLoS One. 2020 Jun 16;15(6):e0234720. doi: 10.1371/journal.pone.0234720 (PMC7297313; doi:10.1371/journal.pone.0234720)
Supplement: S2 Table — (DOCX) [file pone.0234720.s002.docx]

**Appendix Table 2. Search terms from each database**

| **Database** | **Search terms**  **Articles going into screening(duplicates removed) = 2,557* | **Number of results, total = 3,773** |
| --- | --- | --- |
| **PubMed** | (“HIV”[MeSH Terms] OR “human immunodeficiency virus”[All Fields] OR “acquired immunodeficiency syndrome”[All Fields], OR “AIDS”[All Fields]) AND (“prevention"[All Fields] OR "HIV prevention”[All Fields] OR “condom use”[All Fields] OR “HIV testing”[All Fields] OR “STI testing”[All Fields] OR “STD testing”[All Fields] OR “reducing number of sexual partners”[All Fields] OR “injection drug use”[All Fields] OR “PrEP”[All Fields] OR “circumcision”[All Fields] OR “concurrency”[All Fields] OR “intercourse”[All Fields]) AND (“religion”[MeSH Terms] OR “faith”[All Fields] OR “spirituality”[All Fields] OR “spiritual”[All Fields] OR “moral”[All Fields] OR “theological”[All Fields] OR “holy”[All Fields] OR “sacred”[All Fields] OR “doctrinal”[All Fields] OR “church”[All Fields] OR “worship”[All Fields] OR “pray”[All Fields] OR “prayer”[All Fields] OR “religious denomination”[All Fields]) | English, 2000-2020: 811 |
| **Embase** | ((HIV* or human immunodeficiency virus* or acquired immunodeficiency syndrome* or AIDS*) and (prevention* or HIV prevention* or condom use* or HIV testing* or STI testing* or STD testing* or reducing number of sexual partners* or injection drug use* or PrEP* or circumcision* or concurrency* or intercourse*) and (religion* or faith* or spirituality* or spiritual* or moral* or theological* or holy* or sacred* or doctrinal* or church* or worship* or pray* or prayer* or religious denomination*)).mp. | English, 2000-2020: 2,010 |
| **Academic Search Premier** | (AB(“HIV”[MeSH Terms] OR “human immunodeficiency virus” OR “acquired immunodeficiency syndrome” OR “AIDS”) OR TI(“HIV”[MeSH Terms] OR “human immunodeficiency virus” OR “acquired immunodeficiency syndrome” OR “AIDS”)) AND (AB(“prevention” OR “HIV prevention” OR “condom use” OR “HIV testing” OR “STI testing” OR “STD testing” OR “reducing number of sexual partners” OR “injection drug use” OR “PrEP” OR “circumcision” OR “concurrency” OR “intercourse”) OR TI(“prevention” OR “HIV prevention” OR “condom use” OR “HIV testing” OR “STI testing” OR “STD testing” OR “reducing number of sexual partners” OR “injection drug use” OR “PrEP” OR “circumcision” OR “concurrency” OR “intercourse”)) AND (AB(“religion”[MeSH Terms] OR “faith” OR “spirituality” OR “spiritual” OR “moral” OR “theological” OR “holy” OR “sacred” OR “doctrinal” OR “church” OR “worship” OR “pray” OR “prayer” OR “religious denomination”) OR TI(“religion”[MeSH Terms] OR “faith” OR “spirituality” OR “spiritual” OR “moral” OR “theological” OR “holy” OR “sacred” OR “doctrinal” OR “church” OR “worship” OR “pray” OR “prayer” OR “religious denomination”)) | English, 2000-2020: 224 |
| **Web of Science** | (“HIV”[MeSH Terms] OR “human immunodeficiency virus” OR “acquired immunodeficiency syndrome” OR “AIDS”) AND (“prevention” OR “HIV prevention” OR “condom use” OR “HIV testing” OR “STI testing” OR “STD testing” OR “reducing number of sexual partners” OR “injection drug use” OR “PrEP” OR “circumcision” OR “concurrency” OR “intercourse”) AND (“religion”[MeSH Terms] OR “faith” OR “spirituality” OR “spiritual” OR “moral” OR “theological” OR “holy” OR “sacred” OR “doctrinal” OR “church” OR “worship” OR “pray” OR “prayer” OR “religious denomination”) | English, 2000-2020: 421 |
| **Sociological Abstracts** | (AB(“HIV”[MeSH Terms] OR “human immunodeficiency virus” OR “acquired immunodeficiency syndrome” OR “AIDS”) OR TI(“HIV”[MeSH Terms] OR “human immunodeficiency virus” OR “acquired immunodeficiency syndrome” OR “AIDS”)) AND (AB(“prevention” OR “HIV prevention” OR “condom use” OR “HIV testing” OR “STI testing” OR “STD testing” OR “reducing number of sexual partners” OR “injection drug use” OR “PrEP” OR “circumcision” OR “concurrency” OR “intercourse”) OR TI(“prevention” OR “HIV prevention” OR “condom use” OR “HIV testing” OR “STI testing” OR “STD testing” OR “reducing number of sexual partners” OR “injection drug use” OR “PrEP” OR “circumcision” OR “concurrency” OR “intercourse”)) AND (AB(“religion”[MeSH Terms] OR “faith” OR “spirituality” OR “spiritual” OR “moral” OR “theological” OR “holy” OR “sacred” OR “doctrinal” OR “church” OR “worship” OR “pray” OR “prayer” OR “religious denomination”) OR TI(“religion”[MeSH Terms] OR “faith” OR “spirituality” OR “spiritual” OR “moral” OR “theological” OR “holy” OR “sacred” OR “doctrinal” OR “church” OR “worship” OR “pray” OR “prayer” OR “religious denomination”)) | English, 2000-2020: 307 |
