# Supplementary material for: Religion, faith, and spirituality influences on HIV prevention activities: A scoping review
Source: PLoS One. 2020 Jun 16;15(6):e0234720. doi: 10.1371/journal.pone.0234720 (PMC7297313; doi:10.1371/journal.pone.0234720)
Supplement: S3 Table — (DOCX) [file pone.0234720.s003.docx]

| **Appendix Table 3. Quality Assessment of Selected Studies (n = 29)** | | | | | | | |
| --- | --- | --- | --- | --- | --- | --- | --- |
| **Author** | **Year** | **(1) Methods for selecting study participants** | **(2) Methods for measuring exposure and outcome variables** | **(3) Design specific source of bias** | **(4) Method of control confounding** | **(5) Statistical methods** | **(6) Other biases (i.e. conflict of interest and disclosure of funding sources)** |
| Avants, et al. | 2001 | - | + | - | + | + | + |
| McCree, et al. | 2003 | + | + | + | + | + | + |
| Agadjanian | 2005 | ? | + | + | + | + | + |
| Margolin, et al. | 2006 | + | + | + | + | + | + |
| Agha, et al. | 2006 | + | + | + | + | + | + |
| Cerqueira-Santos, et al. | 2008 | + | + | + | - | + | + |
| Perez-Jimenez, et al | 2009 | - | N/A | N/A | N/A | N/A | + |
| Coleman, et al. | 2009 | - | + | - | - | + | + |
| Trinitapoli, et al. | 2009 | + | + | + | + | + | + |
| Wu, et al. | 2010 | + | - | - | - | + | - |
| Agardh, et al | 2010 | - | + | - | + | + | + |
| Berkeley-Patton, et al | 2010 | - | + | + | - | - | + |
| Agardh, et al | 2011 | - | + | - | + | + | + |
| Muula, et al. | 2011 | + | + | + | + | + | - |
| Trinitapoli, et al | 2011 | + | + | + | + | + | + |
| Mash, et al. | 2012 | + | + | + | + | + | + |
| Wingood, et al. | 2013 | + | + | + | + | + | + |
| Szaflarski, et al | 2013 | + | + | + | + | + | + |
| Kagimu, et al | 2013 | - | + | - | - | + | - |
| Downs, et al. | 2013 | - | N/A | N/A | N/A | N/A | + |
| Eriksson, et al. | 2014 | - | - | - | + | + | + |
| Ezeanolue, et al. | 2015 | + | + | + | + | + | + |
| Stewart, et al. | 2016 | - | + | - | ? | + | + |
| Derose, et al. | 2016 | + | + | + | + | + | + |
| Nelson, et al. | 2017 | + | + | + | + | + | + |
| Williams, et al | 2018 | + | + | + | + | + | + |
| Ransome, et al | 2018 | + | + | + | + | + | + |
| Berkley-Patton, et al | 2019 | - | + | + | + | + | + |
| Jemmott, et al | 2020 | + | + | + | + | + | + |
| **Note.** Low risk of bias: +; High risk of bias: -; Unclear: ?; Not applicable: N/A | | | | | | | |
