# Supplementary material for: Religion, faith, and spirituality influences on HIV prevention activities: A scoping review
Source: PLoS One. 2020 Jun 16;15(6):e0234720. doi: 10.1371/journal.pone.0234720 (PMC7297313; doi:10.1371/journal.pone.0234720)
Supplement: S4 Table — (DOCX) [file pone.0234720.s004.docx]

| **Appendix Table 4. Religion, faith, and spirituality measures along with direction of associations and mechanisms among the studies included (n = 29)** | | | | | | | | | | | | |
| --- | --- | --- | --- | --- | --- | --- | --- | --- | --- | --- | --- | --- |
|  | **Measures of religiosity, faith, and spirituality** | | | | | **Religious denomination** | | | | |  | **Mechanism** |
| **References** | **Publication Date** | **Attendance** | **Religious scale** | **Spirituality and subjective religiosity** | **Influence on behavior** | **Catholic** | **Muslim** | **Protestant** | **Christian** | **Other** | **Direction of association** |  |
| Avants, et al. | 2001 |  |  | X |  |  |  |  |  |  | 1 |  |
| McCree, et al. | 2003 |  | X |  |  |  |  |  |  |  | 1 | Social influence, beliefs and values related to sexuality |
| Agadjanian | 2005 | X |  |  |  | X |  | X | X |  | 2 | Behavioral norms, beliefs and values related to sexuality |
| Margolin, et al. | 2006 | X |  |  |  |  |  |  |  |  | 1 |  |
| Agha, et al. | 2006 |  |  |  |  | X |  |  | X | X | 2 |  |
| Cerqueira-Santos, et al. | 2008 |  | X |  |  |  |  |  |  |  | 4 |  |
| Perez-Jimenez, et al | 2009 |  |  |  |  |  |  |  |  |  | 3 | Social influence, behavioral norms |
| Coleman, et al. | 2009 |  | X |  |  |  |  |  |  |  | 1 |  |
| Trinitapoli, et al. | 2009 | X |  |  | X | X | X | X | X |  | 1 | Beliefs and values related to sexuality |
| Wu, et al. | 2010 |  |  |  | X |  |  |  |  |  | 1 | Social influence, education, behavior norms, beliefs and values related to sexuality |
| Agardh, et al | 2010 |  |  |  | X |  |  |  |  |  | 3 | Social influence |
| Berkeley-Patton, et al | 2010 | X |  |  | X |  |  |  |  |  | 1 | Social influence, social organization or support, education |
| Agardh, et al | 2011 |  |  |  | X |  |  |  |  |  | 3 | Social influence |
| Muula, et al. | 2011 |  |  |  |  | X | X | X | X | X | 4 |  |
| Trinitapoli, et al | 2011 |  |  |  |  | X | X | X | X | X | 3 | Social influence, social organization & support, behavior norms, beliefs and values related to sexuality, education |
| Mash, et al. | 2012 | X |  |  | X |  |  |  |  |  | 3 | Social influence, social organization and social support, behavior norms, education |
| Wingood, et al. | 2013 | X |  |  |  |  |  |  |  |  | 1 | Social influence, social organization or support, behavior norms, beliefs and values related to sexuality, education |
| Szaflarski, et al | 2013 |  |  |  |  | X | X | X |  | X | 1 | Social and organizational support, education |
| Kagimu, et al | 2013 | X |  |  | X |  |  |  |  |  | 1 | Social influence, beliefs and values related to sexuality |
| Downs, et al. | 2013 |  |  |  | X |  |  |  |  |  | 2 | Social influence, behavioral norms |
| Eriksson, et al. | 2014 |  |  |  |  | X |  |  | X | X | 1 | Behavioral norms |
| Ezeanolue, et al. | 2015 | X |  |  |  |  |  |  |  |  | 1 | Social organization or support |
| Stewart, et al. | 2016 |  |  |  | X |  |  |  |  |  | 3 | Social influence, beliefs and values related to sexuality |
| Derose, et al. | 2016 | X |  |  |  | X |  |  | X |  | 1 | Behavior norms, beliefs and values related to sexuality, education |
| Nelson, et al. | 2017 |  | X |  | X |  |  |  |  |  | 3 | Social influence, behavioral norms, beliefs and values related to sexuality |
| Williams, et al | 2018 |  |  |  | X |  |  |  |  |  | 4 | Behavioral norms, beliefs and values related to sexuality |
| Ransome, et al | 2018 | X |  |  | X |  |  |  |  |  | 1 | Social influence, behavioral norms, beliefs and values related to sexuality |
| Berkley-Patton, et al | 2019 | X | X |  |  |  |  |  |  |  | 1 | Social influence,  education, and behavioral norms, beliefs and values related to sexuality |
| Jemmott, et al | 2020 | X |  |  | X |  |  |  |  |  | 4 | Beliefs and values related to sexuality |

**Note.** Direction of association (1 = Positive/protective; 2 = negative; 3 = mixed results in the same study; 4 = insufficient information to determine an association, and neutral or null findings)

Mechanisms include the following broad categories (1 = behavioral norms; 2 = social organization or support; 3 = social influence; 4 = education; 5 = beliefs and values related to sexuality; 6 = circumcision; 7 = alcohol use)

When denomination is included, this means the study compared one religious tradition to another. The X does not denote that the study is exclusively among that religious tradition.
