# Supplementary figures and images for: Religion, faith, and spirituality influences on HIV prevention activities: A scoping review
Source: PLoS One. 2020 Jun 16;15(6):e0234720. doi: 10.1371/journal.pone.0234720 (PMC7297313; doi:10.1371/journal.pone.0234720)

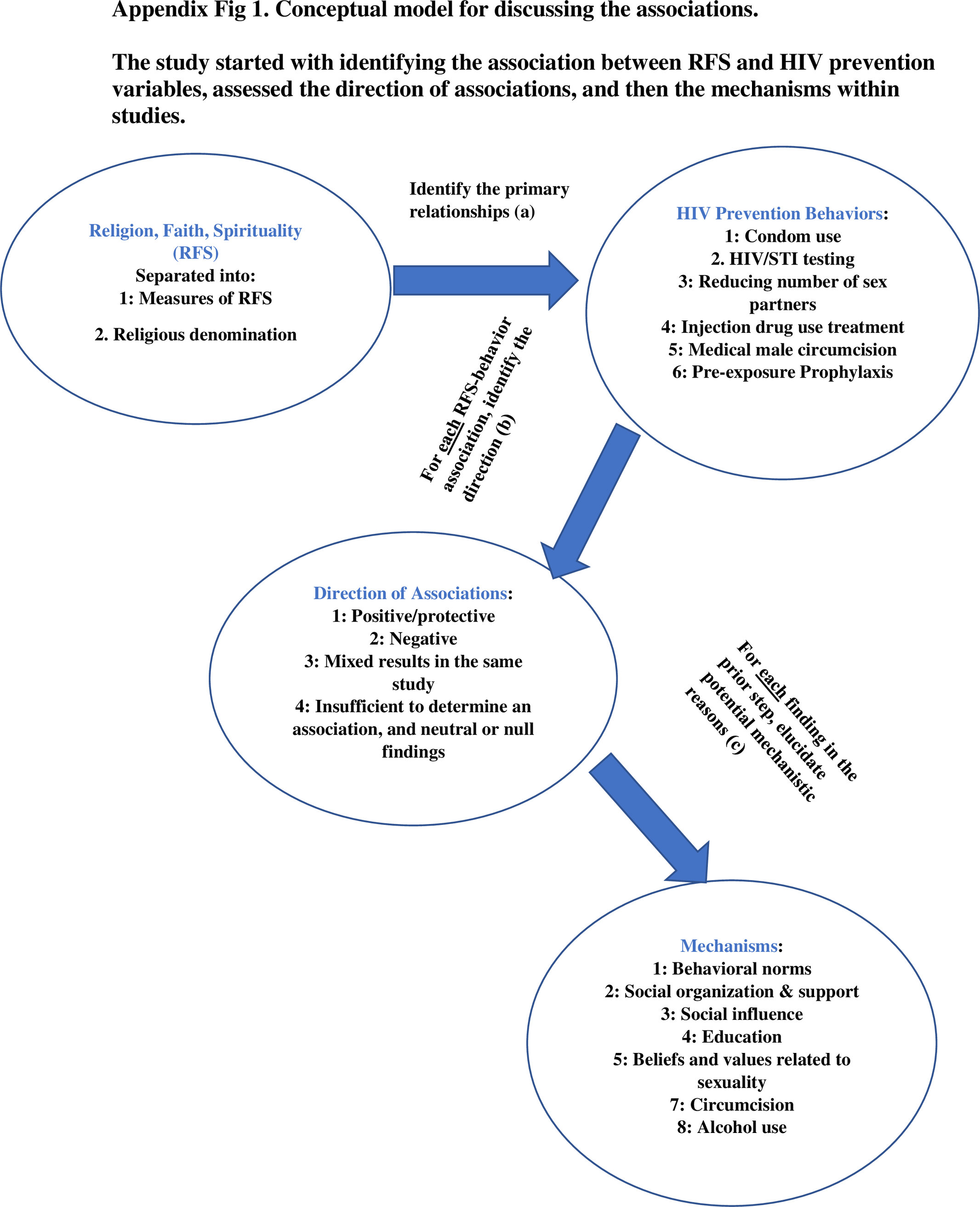

Supplement: S1 Fig — (TIF) [file pone.0234720.s005.tif]

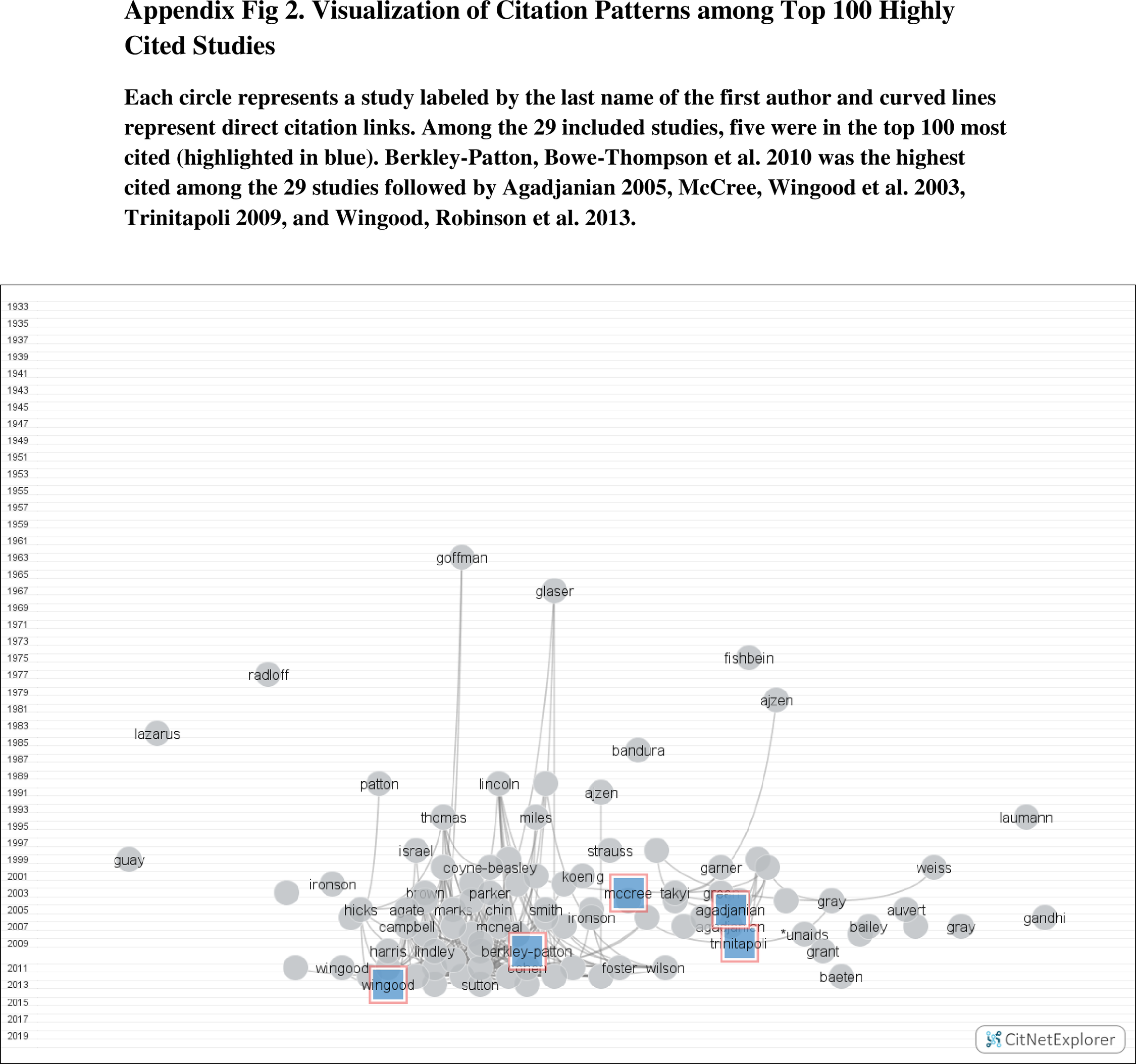

Supplement: S2 Fig — (TIF) [file pone.0234720.s006.tif]
